# Understanding the patient experience of chronic kidney disease stages 2–3b: a qualitative interview study with Kidney Disease Quality of Life (KDQOL-36) debrief

**DOI:** 10.1186/s12882-022-02826-3

**Published:** 2022-06-01

**Authors:** Anna Rydén, Stephen Nolan, Joshua Maher, Oren Meyers, Anna Kündig, Magnus Bjursell

**Affiliations:** 1 Patient Centered Science, Cardiovascular, Renal and Metabolism (CVRM), Biopharmaceuticals R&D, AstraZeneca, Gothenburg, Sweden; 2 Global Medical Affairs, BioPharmaceuticals Medical, AstraZeneca, Cambridge, UK; 3 IQVIA, Reading, UK; 4 IQVIA, New York, USA; 5 Late-Stage Development, Cardiovascular, Renal, and Metabolism (CVRM), BioPharmaceuticals R&D, AstraZeneca, Gothenburg, Sweden; 6 Present address: Global Medical Affairs, Cardiovascular, Renal, and Metabolism (CVRM), BioPharmaceuticals Medical, AstraZeneca, Gothenburg, Sweden

**Keywords:** Chronic kidney disease (CKD), Conceptual model, Symptoms, Health-related quality of life (HRQoL), Kidney Disease Quality of Life 36-item instrument (KDQOL-36), Patient experience, Patient-reported outcome (PRO)

## Abstract

**Background:**

Qualitative patient interviews and patient-reported outcome instruments are important tools to understand the patient experience of disease. The aim of this study was to use patient interviews to identify concepts relevant and important to patients living with chronic kidney disease (CKD) stages 2–3b, develop a comprehensive conceptual model of the patient experience and debrief the Kidney Disease Quality of Life 36-item instrument (KDQOL-36) for patients with CKD stages 2–3b.

**Methods:**

Concept elicitation interviews were conducted with patients with CKD stages 2–3b to identify signs/symptoms and impacts most relevant and important to patients (i.e., ‘salient’ concepts) and develop a conceptual model for the disease. Based on the salient concepts identified in the interviews, new items were proposed to supplement the KDQOL-36. Cognitive debriefing was performed to evaluate the KDQOL-36 and the additional items.

**Results:**

A total of 31 patients were interviewed in this study (22 for concept elicitation and 15 for cognitive debriefing). The interviews identified 56 concepts (33 signs/symptoms and 23 impacts), 17 of which had not been identified in a previous literature review. Four signs/symptoms (‘fatigue/lack of energy/tiredness’, ‘sleep problems’, ‘increased urination [including nocturia]’ and ‘swelling in legs/ankles/feet’) and two impacts (‘anxiety/worry’ and ‘general negative emotional/mental impact’) were identified as salient. Of the salient signs/symptoms, three were not covered by the KDQOL-36 (sleep problems, increased urination and swelling in legs/ankles/feet) and were represented during cognitive debriefing interviews through four additional items (trouble falling asleep, trouble staying asleep, increased urination [including nocturia] and swelling in legs/ankles/feet) generated in the style of the KDQOL-36. All patients found the KDQOL-36 plus the four additional items relevant, and the majority found them clear.

**Conclusions:**

By identifying previously unknown concepts and augmenting the understanding of which are most important to patients, a comprehensive conceptual model was developed for patients who have CKD stages 2–3b. This study also demonstrates the suitability of the KDQOL-36 for patients who have CKD stages 2–3b and provides suggestions for how the instrument could be further developed to more comprehensively capture patient experience.

**Supplementary Information:**

The online version contains supplementary material available at 10.1186/s12882-022-02826-3.

## Background

Chronic kidney disease (CKD) is a global health issue affecting 840 million individuals worldwide and is expected to be the fifth leading cause of mortality by 2040 [1–3]. An estimated glomerular filtration rate (eGFR) of less than 60 mL/min per 1.73 m^2^ and/or urine albumin to creatinine ratio greater than 30 mg/g for more than 3 months are typically used in diagnosis and staging of CKD [4].

The severity and progression of CKD is divided into five stages (1–5) based on eGFR. At stage 1 there is evidence of kidney disease, but kidney function is still normal, and by stage 5 (end-stage kidney disease [ESKD]) kidney function is no longer sufficient to sustain essential body functions [4]. Stage 3 is divided into 3a and 3b, indicating ‘mildly to moderately decreased’ and ‘moderately to severely decreased’ activity, respectively [5].

CKD is associated with several comorbidities, including diabetes, hypertension, anaemia, hyperuricaemia and gout, and cardiovascular diseases including heart failure [6–9]. The combined effect of such conditions can cause increased morbidity and mortality [1, 4, 10]. As patients’ CKD progresses there is an increased risk of cardiovascular events, adverse kidney outcomes and death [11–15]. It is also recognized that patients experience mental health conditions and symptoms, including fatigue and sleep disorders, as consequences of CKD [6, 16]. The combination of physical, mental and social impacts of CKD on patients can negatively affect their quality of life [6, 16, 17].

A comprehensive understanding of patient experience can improve dialogue between patients and clinicians, allowing more informed treatment decisions and, consequently, better patient care [18–20]. Direct patient reporting can help identify previously unrecognized symptoms and highlight aspects of a disease or therapy that patients feel have the greatest impact on their lives [21, 22].

Monitoring patient experience can also help improve clinical trial design and more holistically capture the effects of investigational therapies for CKD [19, 20]. The severity and range of impacts of a disease or treatment can be monitored over time using patient-reported outcome (PRO) instruments. Overlooking patient experience risks missing burdensome impacts that were not captured by the clinical trial adverse event reporting [23, 24]. Benefits of understanding patient experiences include improved quality of care, better health-related quality of life (HRQoL), increased adherence to treatment, reduced hospitalization and increased overall survival [25–29].

The importance of understanding patient experience is increasingly recognized in regulatory and clinical domains because the information enriches traditional efficacy and safety data by giving a more complete understanding of the effects of disease and treatment [18–20, 30–32].

There are currently limited data covering the signs/symptoms and life impacts experienced by patients with CKD stages 2–3b. A lack of overt symptoms specific to the early stages of CKD mean there is an extremely low diagnosis rate, so opportunities for early intervention are missed [33]. Improved understanding of the signs/symptoms and impacts of CKD stages 2–3b could help improve recognition and management of the disease thus slowing progression and preserving patient HRQoL.

PROs can be used to record experiences, including signs, symptoms and functional impacts, of diseases or treatments directly from the patients, without interpretation by clinicians or anyone else [31]. In clinical practice, information from PROs aids understanding of the patient perspective and helps optimize care. Qualitative data collected directly from target clinical study populations of a sufficient sample size, such as through patient interviews, are essential to help inform the design and improvement of PRO instruments by ensuring their content validity [18, 20]. Concepts that are most relevant and important to patients (i.e., ‘salient’ concepts) can be identified based on how many patients experience the sign, symptom or impact, and how much it interferes with patients’ lives. The content validity of PRO instruments is vital to ensure all appropriate concepts are captured, including those most relevant and important to target patients [18, 20, 34].

A previous literature review found that the Kidney Disease Quality of Life 36-item instrument (KDQOL-36) had strong coverage of concepts important for understanding the experience of patients living with CKD [35]. The KDQOL-36 includes an HRQoL measure and three scales that measure the burden, symptoms, problems and effects of kidney disease. As such, the KDQOL-36 explores not only the signs and symptoms of kidney disease, but also the life impacts associated with the disease, including physical and mental states, as well as its effects on HRQoL [36, 37]. However, there has been limited evaluation of the content validity of the KDQOL-36. Further analysis is required to ensure patients with CKD deem all included concepts to be relevant. Input from patients could also be used to update the KDQOL-36 with new concepts missing from the instrument or develop those already present. Such changes could help improve the validity of the KDQOL-36.

The aim of this study was to use patient interviews to identify concepts relevant and important to patients living with CKD stages 2–3b and to develop a comprehensive conceptual model covering their experience. This study also aimed to use information collected from the interviews to help evaluate the content validity of the KDQOL-36 and novel concepts identified during the interviews, as well as confirm whether the instrument is robust for patients with CKD stages 2–3b.

## Methods

### Study design and oversight

**Fig. 1:**
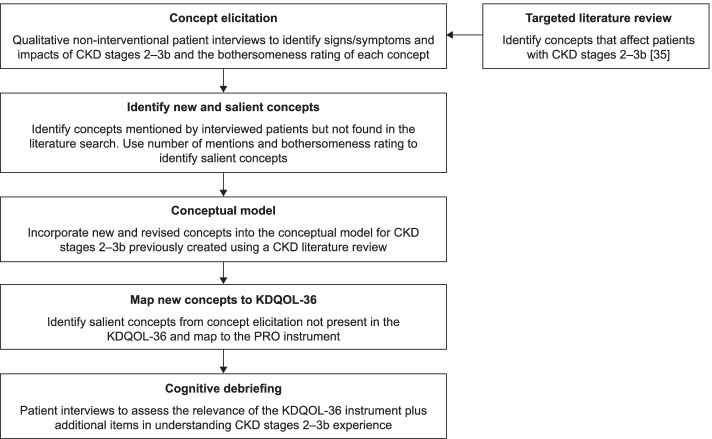
Study flow diagram. (CKD, chronic kidney disease; KDQOL-36, Kidney Disease Quality of Life 36-item instrument; PRO, patient-reported outcome)

Qualitative concept elicitation interviews were conducted to develop a conceptual model capturing the signs/symptoms and impacts most relevant and important to patients with CKD stages 2–3b. The identified salient concepts were then mapped to the KDQOL-36 to evaluate the content validity of the KDQOL-36. New items were created for those salient concepts that did not have corresponding items within the KDQOL-36. The KDQOL-36 plus additional items were then tested by patients through cognitive debriefing interviews to find out whether they were relevant and clear for patients with CKD stages 2–3b. An overview of the study design is given in Fig. 1. This study has been reported following guidance provided by the Consolidated Criteria for Reporting Qualitative Studies (COREQ) checklist [38].

### Study population

Patients were recruited with assistance from the patient recruitment agency Global Perspectives. Outreach techniques used included email, social media and physician contact. All potential participants provided informed consent online and then answered several screening questions to confirm their eligibility. The qualitative interview research protocol, interview guide, and all patient communication documents were reviewed by the New England Institutional Review Board for ethics compliance.

Eligible patients were aged 18 years or older, had a confirmed diagnosis of stage 2–3b CKD (based on patients returning confirmation of diagnosis forms completed by their treating physicians, or confirmation through medical records) and were not receiving dialysis treatment at the time of interview. Patients with a type 2 diabetes mellitus diagnosis could participate in the study but were to make up no more than 50% of the study population to ensure a range of CKD aetiologies were captured. A summary of patient eligibility criteria is given in Table 1. Patients were also asked to provide, where possible, their urine albumin to creatinine ratio and what medications/treatments they were receiving (diuretics, renin–angiotensin–aldosterone system blocking agents, angiotensin II receptor blockers [ARBs] and sodium-glucose co-transporter-2 [SGLT2] inhibitors).

**Table 1:** Qualitative patient interview eligibility criteria

	**Inclusion criteria**	**Exclusion criteria**
Demographics	• Male or female ≥ 18 years at the time of signing informed consent • Speak fluent English • Able to hold a 60-min conversation over the telephone independently • Has access to a computer	• < 18 years at the time of signing informed consent • Unable to speak fluent English • Unable to independently hold a 60-min conversation over the telephone • Does not have access to a computer
Diagnosis	• Stage 2/3a/3b CKD. Duration > 3 months • Serum creatinine-based eGFR ≥ 30 and ≤ 89 mL/min/1.73 m^2^ (CKD-EPI)	• No CKD. Stage 1/4 CKD • Serum creatinine-based eGFR < 30 and > 89 mL/min/1.73 m^2^ (CKD-EPI)
Comorbidities	• Type 2 diabetes mellitus (≤ 50% of study population) • Type 1 diabetes mellitus (≤ 5% of study population) • Hyperuricaemia (≤ 30% of study population)	• Type 2 diabetes mellitus (> 50% of study population) • Type 1 diabetes mellitus (> 5% of study population) • Hyperuricaemia (> 30% of study population) • Autosomal dominant or autosomal recessive polycystic kidney disease • Lupus nephritis or ANCA-associated vasculitis • Autoimmune kidney diseases including glomerulonephritis • Congenital urinary tract malformations
Therapy		• Current (or within 90 days) chronic or intermittent haemodialysis or peritoneal dialysis • Receiving cytotoxic therapy, immunosuppressive therapy or other immunotherapy for primary or secondary renal disease within 6 months prior to enrolment • Previously received an organ transplant • NYHA class IV Congestive Heart Failure at the time of enrolment • Myocardial infarction, unstable angina, stroke or transient ischaemic attack within 12 weeks prior to enrolment

*ANCA* anti-neutrophil cytoplasmic antibody, *CKD* chronic kidney disease, *eGFR* estimated glomerular filtration rate, *NYHA* New York Heart Association

### Interviewers

Interviews were conducted by two interviewers (one male, one female; J. Maher [researcher, IQVIA], T. Casanas [market research consultant, independent]) who are trained and experienced in individual patient concept elicitation and cognitive debriefing interviews across a broad range of therapeutic areas. The interviews were the only contact the interviewers had with the interviewed patients. Patients were interviewed one-to-one over the telephone for 60 min following a semi-structured discussion guide. The interview approach used was in line with recommended guidelines provided by the International Society for Pharmacoeconomics and Outcomes Research (ISPOR) Good Research Practices Task Force [18].

### Concept elicitation

The patient interview guide was informed by a targeted literature review (TLR) previously performed to identify signs/symptoms and impacts on HRQoL experienced by patients with CKD across all stages and PRO instruments used in CKD [35]. The TLR was used to construct a preliminary conceptual model of the experience of patients with CKD overall, as well as patient subpopulations with differing CKD causes and severities, and complications of CKD [35].

As part of the interviews, patients were first asked a set of open-ended questions which allowed them to discuss the signs, symptoms and impacts of their CKD spontaneously. Next, the interviewer probed on concepts that were previously identified in the TLR. At this point patients were also asked to rate the bothersomeness of signs, symptoms and impacts they experienced. Bothersomeness was rated using a scale of 0–10 (0 being ‘not at all bothersome’ and 10 being ‘very bothersome’). For any ratings reported as a range (e.g. 6 or 7) or between integers (e.g. 6.5), the highest bothersomeness rating was recorded to capture the worst extent of concept bothersomeness. Within these questions, patients were asked about their first experience of the condition and what initially brought them to their doctor, how their experience may have changed over time, and current signs, symptoms and impacts of the condition and its treatments. Audio recordings of the patient interviews by IQVIA were transcribed verbatim by River Mist Transcription Services. Transcripts were anonymized prior to analysis.

#### Qualitative data analysis

Coding of the verbatim transcripts was performed by J. Maher and S. Bondugula, both of IQVIA. Prior to coding patient interviews, a codebook was designed that captured all signs/symptoms and impacts based on the preliminary conceptual model from the TLR [35]. Transcripts were coded in the order the interviews were conducted using ATLAS.ti software (version 8; ATLAS.ti Scientific software Development GmbH, Berlin, Germany). The first two transcripts were coded by two coders to ensure inter-coder agreement (ICA; defined as > 0.7 using Krippendorf’s C-alpha binary test). For the following transcripts, every fifth transcript and then the final transcript were double coded to ensure consistency between coders and respective ICA scores.

Concepts were categorized into ‘Signs and Symptoms’ and ‘Impacts’ that influence patient daily living and HRQoL. Transcripts were then grouped chronologically into waves of discussion. Each wave contained equal numbers of interview transcripts (five per wave), except the final wave which included fewer remaining transcripts. The frequency of concepts being mentioned was tracked and reported concepts were labelled as ‘spontaneous’ if unprompted and ‘probed’ if identified through aided questions. Some concepts were only identified following coding and not during the interviews themselves; consequently, not all patients who mentioned a concept were probed for a bothersomeness rating, so the number of bothersomeness ratings recorded for a concept may not equal the number of patients who mentioned it.

Saturation of concepts was defined as the point at which additional patient interviews did not contribute new unique concepts or information [18]. Saturation was achieved when a wave of interviews did not identify any new concepts compared with the previous waves. Concepts were deemed salient when they were mentioned by at least 50% of patients and had a mean bothersomeness rating of 5 or higher (on a 0–10 scale).

#### Conceptual model

Based on the interviews, a conceptual model was constructed of the signs/symptoms and impacts experienced by patients with CKD stages 2–3b which led to an update of the preliminary model from the TLR [35]. The signs/symptoms were grouped into seven domains (pain/discomfort, energy-related, sleep-related, gastrointestinal-system-related, urinary-system-related, skin-/hair-/nails-related, and other) and life impacts grouped into five domains (psychological/emotional impact, cognitive impairment and mental impact, social impact, daily living impacts, and other).

### Cognitive debriefing

Cognitive debriefing interviews were used to assess if patients found the PRO instrument items clear to understand and relevant to their disease experience. Patients were debriefed on 35/36 items from the KDQOL-36 (excluding the item related only to patients who were undergoing dialysis) and the additional items developed in the style of KDQOL-36 (including language used, response options and recall period) to address gaps identified in content coverage. Patients were asked to respond based on their experiences in the 4 weeks prior to being interviewed. The patients were asked to complete and evaluate the PRO items during the interview and give feedback on the items’ clarity and relevance to their CKD experience, the appropriateness of the recall period, and any concept they think had been omitted. Interview transcripts were anonymized and used to assess the clarity and relevance of the KDQOL-36 and any additional items.

## Results

### Study population

A total of 31 patients were interviewed in this study. Twenty-two patients were interviewed for concept elicitation. The mean age was 58 years, over half were female (59%) and over four-fifths were white/Caucasian (86%). There was an even split between those diagnosed with CKD stages 2 and 3a (50%) and stage 3b (50%) and roughly a quarter (27%) were diagnosed with type 2 diabetes (Table 2).

**Table 2:** Demographic and clinical characteristics of patients participating in qualitative interviews

Demographic characteristics	Patients	
	**Concept elicitation** **(*N* = 22)**	**Cognitive debriefing** **(*N* = 15)**
**Sex, n (%)**		
Female	13 (59.1)	8 (53.3)
**Age, years, Mean (SD)**	58.5 (10.1)	61.9 (9.0)
**Ethnicity, n (%)**		
White/Caucasian	19 (86.4)	14 (93.3)
African American	2 (9.1)	1 (6.7)
White/Hispanic	1 (4.6)	0
**CKD stage, n (%)**		
2	5 (22.7)	2 (13.3)
3a	6 (27.3)	9 (60.0)
3b	11 (50.0)	4 (26.7)
**Diabetes diagnosis, n (%)**		
Prediabetes	2 (9.1)	2 (13.3)
Type 2 diabetes	6 (27.3)	5 (33.3)
**eGFR level, mean (SD)**	50.5 (17.3)	49.5 (10.5)
**Time since initial CKD diagnosis, n (%)**		
3–6 months	2 (9.1)	0
6–12 months	4 (18.2)	3 (20.0)
> 12 months	5 (22.7)	0
> 3 years	5 (22.7)	4 (26.7)
> 5 years	3 (13.6)	5 (33.3)
> 10 years	3 (13.6)	3 (20.0)

*CKD* chronic kidney disease, *eGFR* estimated glomerular filtration rate, *SD* standard deviation

Fifteen patients were interviewed for cognitive debriefing, including six who had taken part in the concept elicitation interviews. The mean age was 62 years, roughly half were female (53%) and almost all were white/Caucasian (93%). The majority were diagnosed with CKD stage 3a (13% stage 2, 60% stage 3a, 27% stage 3b) and a third (33%) had type 2 diabetes (Table 2). For both interview phases, patients were on a range of medications/treatments including angiotensin-converting enzyme inhibitors, ARBs, diuretics and SGLT2 inhibitors (Supplementary Table 1). As per the exclusion criteria, none of these patients were undergoing dialysis.

### Concept elicitation patient interviews

The interviews identified 56 concepts (33 signs/symptoms and 23 impacts), 17 of which had not been previously identified by the TLR [35]. All signs/symptoms and impacts were mentioned in the first three interview waves, with no new concepts identified in the final two interview waves, showing that concept saturation was achieved. Most concepts (85% [28/33] of signs symptoms and 74% [17/23] of impacts) were mentioned in the first wave. Inclusion of concepts reported as general negative emotional impacts was based on the patients’ own language when it was described as separate to depression or anxiety. Most newly reported signs/symptoms were related to the urinary system (increased urination, unusual urine colour/consistency, kidney infection). Ten of the twelve patients who reported increased urination (including nocturia) were receiving diuretics. Only one of those ten patients linked the symptom to their treatment and a further four patients receiving diuretics did not report increased urination.

Four signs/symptoms (‘fatigue/lack of energy/tiredness’, ‘sleep problems’, ‘increased urination [including nocturia]’ and ‘swelling in legs/ankles/feet’) and two impacts (‘anxiety/worry’ and ‘general negative emotional/mental impact’) were identified as salient. Example patient quotes included *“Getting to sleep, waking up. Kind of a whole mixture of problems of not feeling well rested and feeling tired and dragging and not having enough energy to kind of falling asleep in the afternoon because my body's so tired and rested”*, *“Bubbles in urine – I think I worry about that. Well, that worries me because I don't know what it means”,* and “*But mental and emotional, that's definitely affected me. Because in the back of my mind I'm struggling. It's a major underlying health condition”*. A further selection of patient quotes describing these concepts are given in Table 3. Of the salient signs/symptoms and impacts identified from patient interviews, three (50%) had not been identified in the previous TLR (‘increased urination [including nocturia]’, ‘swelling in legs/ankles/feet’ and ‘general negative emotional/mental impacts’) [35]. In addition, one sign/symptom (frailty/fractures) and two impacts (confusion and unable to self-care) reported in the previous TLR were not mentioned by the current patient population in the interviews [35]. The majority of signs/symptoms (29/33) and impacts (21/23) were reported as greater than 5 on the bothersomeness scale but were mentioned by less than 50% of patients. Several new concepts that were infrequently mentioned ranked highly on the bothersomeness scale, including three signs/symptoms (kidney infection, headache, and weakness) and three impacts (frustration/anger, fear, and body image issues). Some concepts had a broad range of bothersomeness scores reported. In the case of fatigue/lack of energy/tiredness, the average (mean) bothersomeness rating was 7.1 but patients reported scores ranging from 3 to 10.

**Table 3:** Patient quotes representing salient concepts identified during concept elicitation

	**Concept**	**Patient quote**
Signs/symptoms	Fatigue/tiredness/lack of energy	“Even now, as soon as I wake up like extreme fatigue like I just am so tired, and like I said, I mean, I just have never felt like this before.”
		“Getting to sleep, waking up. Kind of a whole mixture of problems of not feeling well rested and feeling tired and dragging and not having enough energy to kind of falling asleep in the afternoon because my body's so tired and rested.”
	Swelling in legs/ankles/feet	“I will tend to get oedema or swelling in my legs, primarily in my left leg, not my right leg.”
		“I was experiencing a lot of foot pain, and that was based on swelling of the ankles.”
	Increased urination (including nocturia)	“I saw times where it's gotten worse. It even got to the point I would wear a pad in my underwear and be scared to go out. I would be scared to go out.”
		“Because sometimes I will get up in the middle of the night. And that would make me go to the bathroom. I'm only 48. I feel old when I have to do that.”
		“I can’t sleep through the night. Getting up to use the bathroom is about four times a night.”
	Sleep problems	“I feel like I haven’t had a good night’s sleep in over two years. I’ve never slept more than two or three hours at a time.”
		“Some nights I wouldn't sleep correctly, and sleep issues and problems are a big part of kidney disease.”
		“I will have really bad insomnia. It’s hard to fall asleep. Or I’ll be asleep, and I’ll just wake up at 2:00 in the morning, ready to go, so it’s very bizarre sleep patterns.”
Impact	General negative emotional/mental impacts	“It’s more emotionally affects me, I think, than physically because I can’t feel that I have CKD.”
		“For me, CKD is more mental and emotional than physical, primarily because, from what I’m dealing with, there’s really not a lot of hope as far as medications go to improve or maintain the kidney function.”
	Anxiety/worry	“There was certainly some anxiety. I still have a son I need to raise. Am I going to be able to take care of him? What’s going to happen to him if I’m not here?”
		“I'm very worried about the numbers going into a stage 3 because my understanding is once you start to decline with the stages, it kind of happens quickly and can be a really fast downward slide.”

*CKD* chronic kidney disease

### Conceptual model of the CKD patient experience

**Fig. 2:**
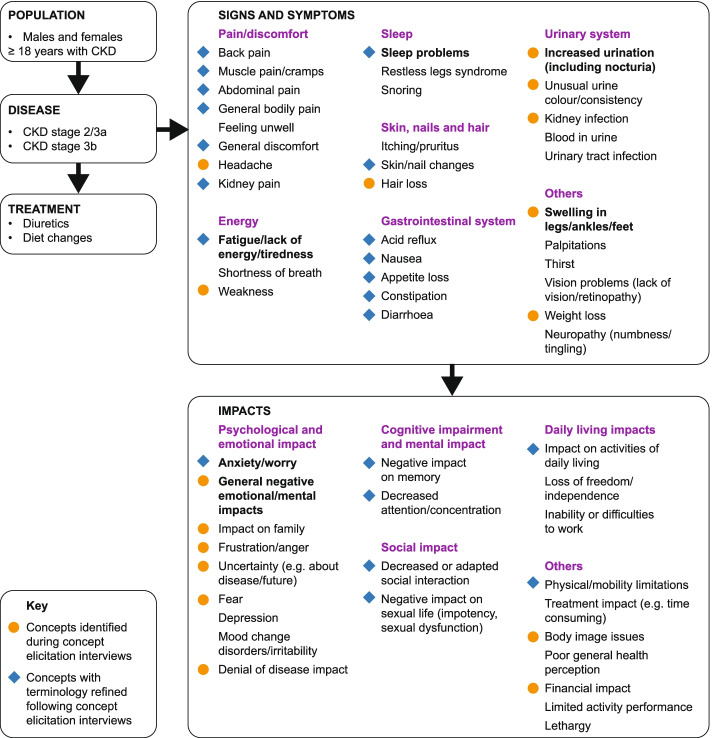
Conceptual model of signs/symptoms and impacts identified in patients with stages 2–3b CKD from patient interviews. Identified signs/symptoms and impacts were grouped into 12 categories, represented by purple headings. New or updated concepts following concept elicitation interviews are indicated by a coloured circle or diamond. Concepts without an orange circle were identified during the TLR [35]. Concepts shown in bold were salient (≥ 50% of patients mentioned the concept and provided an average bothersomeness rating of ≥ 5.0). (CKD, chronic kidney disease; TLR, targeted literature review)

The preliminary conceptual model of signs/symptoms and impacts from the previous TLR [35] was updated to integrate concepts identified in the 22 concept elicitation interviews for CKD stages 2–3b (Fig. 2). As well as highlighting six salient concepts during concept elicitation, the seventeen newly identified concepts (eight signs/symptoms and nine impacts) were added to the conceptual model. Several signs/symptoms and impacts from the preliminary conceptual model were revised to clarify or split/merge concepts to align with the patients’ interview responses. A summary of the conceptual model updates is given in Supplementary Table 2.

### CKD stage

Minor differences were reported for both signs/symptoms and impacts by patients in both CKD groups stages 2/3a and 3b. Patients with stage 3b reported experiencing five symptoms not reported by patients with stage 2/3a, and stage 2/3a reported experiencing four symptoms not reported by patients with stage 3b (Supplementary Table 3). In addition, patients with stage 3b reported experiencing three impacts that were not reported by patients with stage 2/3a and patients with stage 2/3a reported five impacts that were not reported by patients with stage 3b (Supplementary Table 4). More signs/symptoms and impacts reported by patients with stages 2/3a met criteria for salience than those with stage 3b.

### Cognitive debriefing

Three concepts identified as salient in the concept elicitation interviews were not covered by the KDQOL-36 (sleep problems, increased urination [including nocturia] and swelling in legs/ankles/feet). These three concepts were represented during cognitive debriefing interviews through four additional items in the style of the KDQOL-36: “During the past 4 weeks to what extent were you bothered by each of the following?” (The items were listed below the question: “Trouble falling asleep”; “Trouble staying asleep”; “Increased urination”; and “Swelling in legs/ankles/feet”). ‘Sleep problems’ was split into ‘trouble falling asleep’ and ‘trouble staying asleep’ based on patients reporting these separate issues when discussing sleep problems during cognitive debriefing (Table 3). The 35 items from the KDQOL-36 and the 4 additional items were considered relevant by all patients. Of the 35 items from the KDQOL-36, patients found 28 items (80%) clear, 6 items (17%) had minor comments around clarity and 1 item (3%) had a major comment around clarity. All patients found the four additional items to be clear, apart from one patient who thought it might be helpful to indicate that these items are also specifically relating to a patient’s CKD rather than general health. Three patients also reported it being unclear whether items 17–25 (including muscular soreness, chest pains, dry skin and faintness) were related specifically to their CKD and were not in general or due to another disease. Item 28 (relating to the impact of CKD on fluid restriction) caused the most significant confusion among patients because four patients misunderstood it as ‘fluid retention’ and an additional patient reported initially reading it as ‘fluid retention’ before re-reading and understanding what the question meant. Almost all (95%) of the KDQOL-36 items (that is, 35 of the 36 original KDQOL-36 items plus the 4 additional items identified in the current study) were considered by patients to have an appropriate recall period of 4 weeks. This was considered too short for two items (Item 1: ‘In general would you say your health is’ and Item 4: ‘Accomplished less than you would like’) by two patients who proposed a 3-month recall period instead. Four patients commented on five items in the KDQOL-36 based on the Covid-19 global pandemic, which was ongoing at the time of the interviews. Of these, two items related to social interactions and travel, which had been reduced during the global restrictions, one related to ability to work and two related to physical and mental health. The patients reported that these questions were relevant and clear and would be so during ‘normal’ times. Of the interviewed patients, ten suggested twelve concepts that could potentially be added. However, those raised were already covered either during concept elicitation interviews and not identified as salient or by existing items in the KDQOL-36.

## Discussion

Through concept elicitation interviews, this study identified 56 concepts (33 signs/symptoms and 23 impacts) reported by patients with CKD stages 2–3b. The preliminary conceptual model was generated based on literature only, and then augmented using qualitative concept elicitation interviews to further identify and refine relevant concepts. The KDQOL-36 with four extra items added (based on salient concepts identified during concept elicitation) was found to be clear and relevant as a PRO instrument for patients with CKD stages 2–3b.

PRO instruments must be easily understood and include all concepts deemed relevant and important to patients so that these instruments can fully capture the patient experience of disease [18–20]. Three salient concepts (sleep problems, increased urination [including nocturia], swelling in legs/ankles/feet) were identified during concept elicitation but not present in the KDQOL-36. Thus, they were included with the cognitive debriefing as four new items (trouble falling asleep, trouble staying asleep, increased urination, swelling in legs/ankles/feet). The identification of these new concepts demonstrate that it could be beneficial to add exploratory concepts to existing instruments if not currently covered to gain a more complete understanding of patient experience.

Through cognitive debriefing, patients reported all the items were relevant to their disease experience and the majority found the questions easy to understand. The overall relevance and clarity of the instrument also extended to times during the Covid-19 pandemic and ‘normal’ times. The confusion around Item 28 (‘Fluid restriction?’) could be mitigated by switching its position with Item 29 (concerning ‘Dietary restriction?’). The issue of clarity regarding whether questions were specifically related to their CKD and not in general or due to some other disease, is likely the result of items being presented one at a time during the interviews and so could be resolved by using electronic PROs (ePROs), which can help remind patients of the section header at the beginning of each item. Overall, the KDQOL-36 along with the four additional items tested in the patient interviews are suitable and robust for patients with CKD stages 2–3b.

While the majority of concepts identified in the TLR were confirmed in this study, a range of new concepts were also identified. Although the TLR prioritized qualitative research articles, the current study identified several concepts not found in the TLR [35]. Several concepts identified in the TLR were also revised (split or merged) so they more closely represented the patients’ experiences. These findings demonstrate the value of directly capturing qualitative information from patients.

Several of the concepts identified were recognized as salient, with half being identified only in the concept elicitation patient interviews (four signs/symptoms: fatigue/lack of energy/tiredness, sleep problems, increased urination [including nocturia], and swelling in legs/ankles/feet; and two impacts: anxiety/worry and general negative emotional/mental impacts). The range of reported bothersomeness ratings (from 3 to 10) for fatigue/lack of energy/tiredness demonstrates heterogeneity in signs/symptoms experienced by patients with CKD stages 2–3b as well as possibly their comorbid conditions. The low number of signs/symptoms meeting the present definition of salience (4/33) may be the result of the earlier stages of CKD being less symptomatic. In the case of the low number of salient impacts reported (2/23), half of the patients interviewed received their diagnosis more than 5 years ago, so they may have become used to how CKD impacts their lives [39] or it may have been the result of the low sign/symptom bothersomeness rating. However, many signs/symptoms and impacts were highly bothersome despite being reported by fewer individuals suggesting patients could be experiencing the same signs/symptoms or impacts at different severities or finding similar experiences easier or harder to manage. This demonstrates the variety of experiences for patients with CKD stages 2–3b.

The most commonly reported salient sign/symptom and impact were fatigue/lack of energy/tiredness and general negative emotional/mental impacts, respectively. Both fatigue and impacts on mental health are known to affect patients with many different chronic conditions, including cancer and heart failure, as well as non-dialysis-dependent CKD [40–44]. Experiencing nocturia and mental health conditions such as anxiety/worry and general negative emotional/mental impacts associated with CKD can contribute to sleep disturbances resulting in fatigue/lack of energy/tiredness [45]. Physical changes to an individual’s body, such as swelling in the legs/ankles/feet, could also limit patient mobility as well as negatively impact mental health and ability to work or socialize. Patients with more severe signs/symptoms and associated impacts are more likely to have reduced HRQoL, faster progression through CKD stages to ESKD, increased hospital admissions, and greater risk of death [42, 44, 46–48].

Several concepts identified during concept elicitation were related to the urinary system, including roughly half of patients reporting the salient sign/symptom increased urination (including nocturia). With 10 out of the 12 patients who reported this sign/symptom receiving diuretic treatment for their CKD, it is possible the increased urination is the result of the CKD-related treatment rather than the disease itself. However, the concept was kept and included in the KDQOL-36 cognitive debriefing because it was still recognized as a major part of the CKD disease experience for the majority of patients interviewed. The inclusion of nocturia means this concept can also be linked to sleep problems.

With a focus on understanding patient experience of CKD across stages 2–3b, only minor differences were reported for signs/symptoms and impacts between patients with stages 2/3a and 3b CKD. The patients interviewed experienced similar comorbidities and received similar treatments overall, so it is unlikely these influenced any differences in the results. The differences instead likely reflect the diversity of the early-to-mid-stage population. The number of signs/symptoms and impacts reported by patients with CKD stages 2–3b also indicates that the patients with early-stage CKD do experience a range of symptoms that impact their lives [1, 49]. It is also important to recognize that differences in time since diagnosis may influence the number of signs/symptoms and impacts reported. Patients with a longer time since diagnosis may have had longer to adjust to their condition and so report fewer signs/symptoms and impacts or report them as less severe than individuals with a shorter time since diagnosis [39].

Earlier stages of CKD are known for having nonspecific symptoms which lead to a particularly low diagnosis rate [33]. Identifying new concepts and recognizing any consistencies in signs/symptoms between patients with CKD stages 2-3b can help create a more complete understanding of patient experience of early-stage CKD. They could be valuable for identifying symptoms of and diagnosing CKD sooner so that treatment can be started quicker and progression of the condition to ESKD slowed so as to preserve patient HRQoL [1, 4]. The need to develop this understanding of the patient experience is demonstrated by the new salient concepts identified in this study that may have gone unrecognized if patients were not directly asked about their experience of CKD.

Heterogeneity and lack of standardization exists across all endpoints in CKD. This is being addressed by the Standardised Outcomes in Nephrology (SONG)-CKD initiative, which will include multiple stakeholders, both healthcare professionals (HCPs) and non-HCPs, and will address PRO instruments [50]. The present study is focused on patients and patient outcomes. This study demonstrates it would likely be beneficial to add exploratory concepts to existing PRO instruments, such as the KDQOL-36, to gain a more complete understanding of patient experience. This could improve understanding of CKD-related symptoms and impacts and how to better support patients, as well as inform the development of novel therapies during clinical trials. As our understanding of kidney disease is continuously evolving, it is important to ensure that clinical outcomes assessment measures evolve in tandem so that we continuously strive to capture the complete patient experience.

### Study limitations

By focusing on disease stages 2–3b the conceptual model is restricted to a portion of the CKD population and so does not develop our understanding of the whole CKD experience, making the study more applicable to early-stage disease. The study population was also skewed towards white/Caucasian patients, with a limited representation of other ethnicities. Some of the concepts recorded by patients could have been comorbidities that were incorrectly attributed to CKD; however, inclusion of the measure of salience should minimize the risk of such anomalies being emphasized in the conceptual model. Finally, during cognitive debriefing there was a disparity between the number of patients recruited with stages 2–3a (73%) and stage 3b (33%). This could have influenced the results indicating suitability of the KDQOL-36 plus additional items for the earlier stages.

## Conclusions

This study demonstrated the need for adding more exploratory concepts to the KDQOL-36 to fully capture the patient experience of CKD stages 2–3b. The interviews identified 17 concepts not identified in a previous TLR, resulting in the preliminary conceptual model being updated, as well as identification of salient concepts that were not covered by items in the KDQOL-36. Ultimately, by investigating patient experience for individuals with CKD stages 2–3b it is possible to develop a more complete understanding of how patients are affected by their conditions which could inform diagnosis and treatment decisions, as well as in clinical trial design by identifying endpoints relevant to patients.

## Supplementary Information

**Additional file 1: Supplementary Table 1. **Treatments received by patients interviewed for concept elicitation and cognitive debriefing. **Supplementary Table 2. **Updates to concepts in conceptual model for CKD stages 2–3b. **Supplementary Table 3. **Reported signs/symptoms and bothersomeness ratings by patients with chronic kidney disease stages 2/3a and 3b. **Supplementary Table 4. **Reported impacts and bothersomeness ratings by patients with chronic kidney disease stages 2/3a and 3b.

## Data Availability

All data generated or analysed during this study are included in this published article (and its supplementary information files).

## Abbreviations

ARB: Angiotensin II receptor blocker
CKD: Chronic kidney disease
COREQ: Consolidated Criteria for Reporting Qualitative Studies
eGFR: Estimated glomerular filtration rate
ePRO: Electronic PRO
ESKD: End-stage kidney disease
HRQoL: Health related quality of life
ISPOR: International Society for Pharmacoeconomics and Outcomes Research
KDQOL-36: Kidney Disease Quality of Life 36-item instrument
PRO: Patient-reported outcome
SGLT2: Sodium-glucose co-transporter-2
TLR: Targeted literature review
