# Supplementary material for: Understanding the patient experience of chronic kidney disease stages 2–3b: a qualitative interview study with Kidney Disease Quality of Life (KDQOL-36) debrief
Source: BMC Nephrol. 2022 Jun 1;23:201. doi: 10.1186/s12882-022-02826-3 (PMC9155979; doi:10.1186/s12882-022-02826-3)
Supplement: Supplementary file 1 — Additional file 1: Supplementary Table 1. Treatments received by patients interviewed for concept elicitation and cognitive debriefing. Supplementary Table 2. Updates to concepts in conceptual model for CKD stages 2–3b. Supplementary Table 3. Reported signs/symptoms and bothersomeness ratings by patients with chronic kidney disease stages 2/3a and 3b. Supplementary Table 4. Reported impacts and bothersomeness ratings by patients with chronic kidney disease stages 2/3a and 3b. [file 12882_2022_2826_MOESM1_ESM.docx]

**Supplementary Tables**

**Supplementary Table 1 Treatments received by patients interviewed for concept elicitation and cognitive debriefing**

| **Treatment** | **Concept elicitation** | | **Cognitive debriefing** | |
| --- | --- | --- | --- | --- |
|  | **Stage 2/3a** | **Stage 3b** | **Stage 2/3a** | **Stage 3b** |
| ACE inhibitor/ARB | **Losartan**  **Other ACEs**  **ARBs (for blood pressure)**  Valsartan | **Edarbi**  Lisinopril | **Unspecified RAAS treatment**  **Unspecified ACE inhibitors**  **Valsartan** | **Lisinopril** |
| Diuretics | Lasix | **Intravenous diuretics**  Hydrochlorothiazide | - | Furosemide |
| Diabetic medication | **Ozempic** | Metformin  Metoprolol | **Unspecified SGLT2 medication** | - |
| Anti-hyperlipidaemic | **Atorvastatin**  **Fenofibrate** | - | - | - |
| Calcium channel blockers | **Verapamil** | **Amlodipine** | **Amlodipine** | - |
| Beta blockers | **Nebivolol**  **Tamsulosin**  **Timolol** | **Nebivolol** | - | - |
| Medication for GIT issues | **Bismuth subsalicylate**  **Omeprazole** | **Stool softener**  **Probiotics**  **Laxatives** | - | - |
| Anti-viral medication | **Entecavir**  **Dolutegravir**  **Bictegravir/emtricitabine/tenofovir alafenamide** | - | **Entecavir**  **Dolutegravir**  **Bictegravir/emtricitabine/tenofovir alafenamide** | - |
| Anti-emetics | - | **Zofran** | - | - |
| NSAIDs | **Aspirin**  Ibuprofen (for pain) | - | - | - |
| Antibiotics | Ciproflaxin (for infection) | **Antibiotics (for UTI and cystic fibrosis)**  Ciproflaxin (for infection) | - | - |
| Thyroid medication | **Levothyroxine** | **Levothyroxine** | **Levothyroxine** | - |
| Biological therapy | **Etanercept (for rheumatoid arthritis)** | - | - | - |
| Medication for anxiety/depression | - | **Sertraline**  **Lorazepam** | **Buspirone** | - |
| Surgeries | Surgery for skin cancer  Basal cell carcinoma resection  Cataract surgery  Glaucoma surgery | Nephrectomy  Gum surgery  Kidney surgery  Bladder tumour excision  Ureter stent | - | - |
| Interventions | History of dialysis | History of dialysis | - | - |
| Supplements | **Vitamin D**  **Multiple vitamins**  **Omega 3**  Phentermine | - | **Vitamins**  **Omega-3** | - |
| Others | **Hydrocortisone (for itching)**  **Loratadine**  **Lorazepam**  **Tacrolimus**  **Sumatriptan** | **Medication for pain syndrome**  **Lotions/creams for itching**  **Lyrica (for restless leg syndrome)**  **Epoetin Alfa**  Epoetin zeta (for anaemia)  Hydroxychloroquine | **Ciclesonide**  **Sumatriptan**  **Tacrolimus** | - |

Treatments that patients were using at the time of the interviews are in bold.

ACE, angiotensin-converting enzyme; ARB, angiotensin receptor blockers; GI, Gastrointestinal tract; NSAID, non-steroidal anti-inflammatory drug; RAAS, renin angiotensin aldosterone system; SGLT2, sodium-glucose co-transporter-2; UTI, urinary tract infection

**Supplementary Table 2 Updates to concepts in conceptual model for CKD stages 2**–**3b**

|  | **Concept** | **Updates** |
| --- | --- | --- |
| Sign/symptoms | Pain | Split   - ‘Pain’ split into six different signs/symptoms based on location including ‘back pain’, ‘muscle pain/cramps’, ‘abdominal pain’, ‘general bodily pain’, ‘general discomfort’ and ‘kidney pain’ |
|  | GI symptoms | Split   - ‘GI symptoms’ was split into the five different signs/symptoms ‘acid reflux’, ‘nausea’, ‘appetite loss’, ‘constipation’ and ‘diarrhoea’ |
|  | Skin/nail changes | Revised   - ‘Skin problems’ was revised to ’skin/nail changes’ |
|  | Sleep problems | Revised   - ‘Poor sleep quality’ was revised to ‘sleep problems’ |
|  | Fatigue/tiredness/lack of energy | Merged   - Three separate symptoms (‘fatigue’, ‘tiredness’ and ‘lack of energy’) merged into ‘fatigue/tiredness/lack of energy’ |
|  | Headache | Newly identified |
|  | Weakness | Newly identified |
|  | Hair loss | Newly identified |
|  | Increased urination (including nocturia) | Newly identified |
|  | Unusual urine colour/consistency | Newly identified |
|  | Kidney infection | Newly identified |
|  | Swelling in legs/ankles/feet | Newly identified |
|  | Weight loss | Newly identified |
|  | Frailty and fractures | Not reported |
| Impact | Negative impact on memory | Revised   - ‘Memory’ was revised to ‘negative impact on memory’ |
|  | Decreased attention/concentration | Revised   - ‘Attention, concentration’ was revised to ‘decreased attention/concentration’ |
|  | Decreased or adapted social interaction | Revised   - ‘Social impact’ was revised to ‘decreased or adapted social interaction’ |
|  | Impact on activities of daily living | Revised   - ‘Difficulty to do regular activities (e.g. housework, using tools)’ was revised to ‘impact on activities of daily living’ |
|  | Physical/mobility limitation | Revised   - ‘Mobility limitation’ was revised to ‘physical/mobility limitation’ |
|  | Anxiety/worry | Merged   - ‘Anxiety’ was merged with worry to form ‘anxiety/worry’ |
|  | General negative emotional/mental impacts | Newly identified |
|  | Frustration/anger | Newly identified |
|  | Negative impact on sexual life (impotency, sexual dysfunction) | Newly identified |
|  | Uncertainty (e.g. about disease/future) | Newly identified |
|  | Denial of disease impact | Newly identified |
|  | Body image issues | Newly identified |
|  | Financial impact | Newly identified |
|  | Fear | Newly identified |
|  | Impact on Family | Newly identified |
|  | Confusion | Not reported |
|  | Unable to self-care | Not reported |

GI, gastrointestinal

**Supplementary Table 3 Reported signs/symptoms and bothersomeness ratings by patients with chronic kidney disease stages 2/3a and 3b**

|  | **Stage 2/3a** | | **Stage 3b** | |
| --- | --- | --- | --- | --- |
| **Symptom** | **Total mentions**  **n (%)** | **Average bothersomeness rating** | **Total mentions**  **n (%)** | **Average bothersomeness rating** |
| Fatigue/lack of energy/tiredness | **8 (73)** | **6.8** | 6 (55) | 7.0 |
| Shortness of breath | 2 (18) | 9.0 | 3 (27) | 4.0 |
| Weakness | 2 (18) | 9.0 | 1 (9) | 6.0 |
| Acid reflux | 1 (9) | 8.0 | 0 (0) | - |
| Constipation | 1 (9) | 6.0 | 2 (18) | 9.5 |
| Diarrhoea | 1 (9) | 6.0 | 0 (0) | - |
| Nausea | 0 (0) | - | 3 (27) | 7.0 |
| Appetite loss | 0 (0) | - | 2 (18) | 6.5 |
| Swelling (legs/ankles/feet) | **6 (55)** | **7.0** | 5 (45) | 6.4 |
| Vision problems (lack of vision, retinopathy) | 2 (18) | 10.0 | 1 (9) | 2.0 |
| Thirst | 2 (18) | 5.0 | 2 (18) | 5.5 |
| Weight loss | 1 (9) | 0.0 | 3 (27) | 5.0 |
| Palpitations | 0 (0) | - | 1 (9) | 6.0 |
| Neuropathy (numbness/tingling) | 0 (0) | - | 1 (9) | 9.0 |
| Back pain | **7 (64)** | **7.4** | 2 (18) | 7.5 |
| Muscle pain/cramps | 4 (36) | 8.0 | 4 (36) | 8.5 |
| General discomfort | 3 (27) | 8.3 | 1 (9) | 0.0 |
| General bodily pain | 2 (18) | 10.0 | 1 (9) | 8.0 |
| Feeling unwell | 2 (18) | 9.0 | 2 (18) | 4.5 |
| Abdominal pain | 2 (18) | 8.0 | 0 (0) | - |
| Headache | 0 (0) | - | 2 (18) | 8.0 |
| Itching/pruritus | 4 (36) | 9.3 | 1 (9) | 5.0 |
| Dry skin/nail changes | 4 (36) | 5.0 | 2 (18) | 3.0 |
| Hair loss | 2 (18) | 7.5 | 1 (9) | 2.0 |
| Sleep problems | **7 (64)** | **9.2** | 6 (55) | 7.7 |
| Restless leg syndrome | 3 (27) | 5.7 | 1 (9) | 4.0 |
| Sleep apnoea | 2 (18) | 5.5 | 1 (9) | 8.0 |
| Increased urination (including nocturia) | **6 (55)** | **7.2** | 5 (45) | 6.0 |
| Urinary tract infection | 3 (27) | 9.0 | 2 (18) | 5.0 |
| Unusual urine colour/consistency | 3 (27) | 2.0 | 5 (45) | 7.5 |
| Kidney infection | 2 (18) | 10.0 | 0 (0) | - |
| Blood in urine (haematuria) | 1 (9) | 4.0 | 2 (18) | 8.0 |

Concepts not mentioned by any patients in a specific stage are in red.

Concepts mentioned by ≥ 50% of patients are in bold.

**Supplementary Table 4 Reported impacts and bothersomeness ratings by patients with chronic kidney disease stages 2/3a and 3b**

| **Impact** | **Stage 2/3a** | | | **Stage 3b** | |
| --- | --- | --- | --- | --- | --- |
|  | **Total mentions n (%)** | | **Average bothersomeness rating** | **Total mentions n (%)** | **Average bothersomeness rating** |
| Decreased attention/concentration | 3 (27) | 7.0 | | 0 (0) | - |
| Negative impact on memory | 2 (18) | 5.5 | | 1 (9) | 2.0 |
| Impact on activities of daily living | 3 (27) | 8.0 | | 1 (9) | 9.0 |
| Loss of freedom/independence | 2 (18) | 8.0 | | 0 (0) | - |
| Ability to work | 1 (9) | 8.0 | | 0 (0) | - |
| Physical/mobility limitations | 4 (36) | 7.7 | | 2 (18) | 7.5 |
| Treatment impact (e.g. time consuming) | 3 (27) | 8.0 | | 4 (36) | 4.0 |
| Limited activity performance | 3 (27) | 5.7 | | 1 (9) | 9.0 |
| Financial impact | 2 (18) | 6.5 | | 2 (18) | 7.0 |
| Vitality | 2 (18) | 3.5 | | 0 (0) | - |
| Body image issues | 0 (0) | - | | 2 (18) | 8.0 |
| Poor health perception | 0 (0) | - | | **7 (64)** | **7.5** |
| General negative emotional/mental impacts | **6 (55)** | **7.7** | | **6 (55)** | **6.0** |
| Anxiety/worry | 5 (45) | 7.5 | | **6 (55)** | **8.0** |
| Impact on family | 5 (45) | 5.7 | | 4 (36) | 6.0 |
| Uncertainty (e.g. about disease/future) | 4 (36) | 7.0 | | 3 (27) | 7.5 |
| Frustration/anger | 3 (27) | 8.0 | | 4 (36) | 9.3 |
| Fear | 2 (18) | 9.5 | | 3 (27) | 8.0 |
| Depression | 2 (18) | 7.0 | | 1 (9) | 9.0 |
| Mood change disorders/irritability | 1 (9) | 0.0 | | 0 (0) | - |
| Denial of disease impact | 0 (0) | - | | 1 (9) | 0.0 |
| Decreased or adapted social interaction | 5 (45) | 6.0 | | 4 (36) | 5.3 |
| Negative impact on sexual life (impotency, sexual dysfunction) | 1 (9) | 6.0 | | 4 (36) | 7.8 |

Concepts not mentioned by any patients in a specific stage are in red.

Concepts mentioned by ≥ 50% of patients are in bold.
